# Humane Slaughter of Edible Decapod Crustaceans

**DOI:** 10.3390/ani11041089

**Published:** 2021-04-11

**Authors:** Francesca Conte, Eva Voslarova, Vladimir Vecerek, Robert William Elwood, Paolo Coluccio, Michela Pugliese, Annamaria Passantino

**Affiliations:** 1 Department of Veterinary Sciences, University of Messina, Polo Universitario Annunziata, 981 68 Messina, Italy; fconte@unime.it (F.C.); mpugliese@unime.it (M.P.); passanna@unime.it (A.P.); 2 Department of Animal Protection and Welfare and Veterinary Public Health, Faculty of Veterinary Hygiene and Ecology, University of Veterinary Sciences Brno, 612 42 Brno, Czech Republic; vecerekv@vfu.cz; 3 School of Biological Sciences, Queen’s University, Belfast BT9 5DL, UK; R.Elwood@qub.ac.uk; 4 Department of Neurosciences, Psychology, Drug Research and Child Health (NEUROFARBA), University of Florence-Viale Pieraccini, 6-50139 Firenze, Italy; paolo.coluccio@unifi.it

**Keywords:** Decapoda, stunning, slaughtering techniques, welfare, legislation

## Abstract

**Simple Summary:**

Decapods respond to noxious stimuli in ways that are consistent with the experience of pain; thus, we accept the need to provide a legal framework for their protection when they are used for human food. We review the main methods used to slaughter the major decapod crustaceans, highlighting problems posed by each method for animal welfare. The aim is to identify methods that are the least likely to cause suffering. These methods can then be recommended, whereas other methods that are more likely to cause suffering may be banned. We thus request changes in the legal status of this group of animals, to protect them from slaughter techniques that are not viewed as being acceptable.

**Abstract:**

Vast numbers of crustaceans are produced by aquaculture and caught in fisheries to meet the increasing demand for seafood and freshwater crustaceans. Simultaneously, the public is increasingly concerned about current methods employed in their handling and killing. Recent evidence has shown that decapod crustaceans probably have the capacity to suffer because they show responses consistent with pain and have a relatively complex cognitive capacity. For these reasons, they should receive protection. Despite the large numbers of crustaceans transported and slaughtered, legislation protecting their welfare, by using agreed, standardized methods, is lacking. We review various stunning and killing systems proposed for crustaceans, and assess welfare concerns. We suggest the use of methods least likely to cause suffering and call for the implementation of welfare guidelines covering the slaughter of these economically important animals.

## 1. Introduction

Global use of decapod crustaceans (such as lobsters, prawns, crayfish and crabs) for human consumption is growing. Many billions of individuals are either wild caught or produced in aquaculture systems each year [1]. Historically, there has been little concern for the welfare of these animals because they were thought to have no ability to experience pain, and simply depended on nociceptive reflexes to escape from noxious stimuli [2]. However, recent studies demonstrated that some responses of decapods cannot be explained as reflexes, and this opens the possibility that they feel pain and need to be protected [1,2,3,4]. By contrast, others [5,6,7,8] argue that the scientific literature on crustacean pain is weak and inconclusive, and there is no requirement to be concerned about their welfare. Thus, there is no consensus about decapods being sentient in terms of feeling pain. However, it is not possible to obtain absolute, objective proof of pain in any individual (humans or animals). Yet it is commonly accepted that we should err on the side of caution in how we treat animals. Following the precautionary principle, some animal taxa receive legal protection in various jurisdictions and contexts, but this is rare for decapods [9].

Here, we focus on the problem of the humane slaughter of decapod crustaceans in the human food chain. We first examine the evidence for and against the experience of pain and potential for suffering in this group. Second, we review the legal position regarding slaughter of these animals. We then review slaughter and stunning methods for decapods and, finally, suggest ways that these animals might be protected from inhumane methods to better protect their welfare.

## 2. Nociception, Pain and Stress in Decapods

Nociception refers to the ability to detect and respond to a noxious stimulus. This is achieved by the responses of nociceptors, which have relatively unspecialized, naked nerve endings that respond to mechanical, thermal, or chemical stimuli or a combination of these. When they fire, they initiate a reflex by which the animal withdraws part or all of its body to avoid further noxious stimuli. Importantly for understanding the complexity of pain, the reflex does not necessarily involve the central nervous system or central processing and does not necessarily produce the emotional experience of pain [10]. Thus, simply noting that an animal avoids noxious stimuli does not enable pain to be inferred.

Nociceptors are conserved and found in most animal groups and have been well-studied in arthropods. For example, numerous studies on *Drosophila* have been key to our understanding the development and functioning of these nerve cells [11]. They have been much less studied in decapods, but they have been inferred from the behavioural responses of these animals and from examination of surface receptors [10]. Decapods respond to mechanical, thermal, and chemical stimuli [7,12], and receptors have been described that respond to those stimuli [7,13]. These receptors may be packaged into cuticular extensions of the exoskeleton called sensilla [14,15,16].

Thus, decapods have the sensory cells to enable the perception of noxious stimuli and they show behavioural responses to those stimuli. However, as noted above, these responses might be nociceptive reflexes and might occur without the need for the complex central processing thought to be required for pain experience. One objection to the possibility of pain in decapods is that they do not have a sufficiently large, complex central nervous system [17]. However, the brains (supraesophageal ganglia) of some decapods are as large as the brains in some fish, and they have a high degree of compartmentalization of function [18]. They have inputs from various sensory systems [18] and arthropod brains allow for remarkably advanced cognitive processes [19]. A second objection about decapods feeling pain is that the brain would have to be homologous to that of humans because the anatomy is fitted to function [17]. That is clearly not the case, however, because many animals with vastly different brain structures have similar abilities despite having brains that are very different to those of humans, for example, vision in cephalopods and decapods [20]. However, whether decapods have brains of sufficient complexity to experience pain has yet to be shown conclusively. 

A consideration of function and evolution might provide insights into the possibility of pain in various taxa. Nociception enables an immediate withdrawal from the stimulus and thus provides important protection for the animal. Why then have some animal taxa evolved the associated negative emotional response that we call pain? No doubt there is a cost of developing the pain system, so what benefit might outweigh that cost? The only convincing argument is that with just reflex responses, without central processing, there will be no memory of the event and no prolonged shift in behaviour to provide protection from further damage, and to allow recovery from injury [20,21]. Thus, if pain can be experienced, we expect non-reflexive, long-term, changes in behaviour and an indication of central processing. 

The behavioural criteria for pain, and experiments designed to test those in decapods, have been reviewed extensively [4,20,22,23,24] and here we aim to be brief. Hermit crabs show a trade-off of shock avoidance with other motivational requirements, indicating that rapid shell-evacuation is the result of a decision that requires central processing rather than reflex [25,26,27]. Shore crabs rapidly learn to avoid entering a dark shelter associated with electric shock and will pay a cost to avoid the shock [28]. Hermit crabs show long-term changes in behaviour following a shock within their shell and become less motivated to retain that shell [27,29,30], a shift that lasts for at least a day [31]. Glass prawns show prolonged rubbing and grooming of a specific antennae that has had a noxious chemical applied, but this is markedly reduced if it had been treated previously with a local anaesthetic [12], and edible, brown crabs show guarding of a wounded area after claw removal [32]. Furthermore, shore crabs hold down just one eye within the protective socket if that eye had been brushed with acid [33] and crabs with a claw injected with formalin shake and attend to that specific appendage and might autotomize it [34]. Thus, decapods show prolonged responses directed towards specific body parts. Crayfish subjected to repeated electric shocks subsequently show signs of anxiety and avoid brightly lit areas; however, avoidance is reduced if they are given a drug designed to reduce anxiety in humans [35,36]. These studies are all consistent with the experience of pain. Importantly, they demonstrate the long-term modification of behaviour likely to enhance fitness.

As pain is aversive and produces such a powerful emotional response, it is expected to trigger stress. Stress is how the body responds to changes that create taxing demands [37]. In vertebrates, it causes a cascade of hormonal changes that leads to the production of adrenal hormones (cortisol and corticosterone), which stimulate the conversion of glycogen to glucose used in the fight-or-flight response. In decapods, stress releases the crustacean hyperglycemic hormone (CHH), which has broadly similar effects to those adrenal hormones in vertebrates [10], or by releasing biogenic amines such as epinephrine and serotonin [38,39].

Rapid stress responses, such as elevated lactate, glucose, and muscular glycogen mobilisation, are seen in edible crabs that have a claw forcefully removed, as in fishery practice, which causes tissue damage [40]. This was not seen in crabs induced to autotomize, which does not cause tissue damage. It is not clear, however, if this was due to pain or the tissue damage per se. However, similar changes occurred when shore crabs were given electric shocks [41]. Repeated electric shocks also caused elevated serotonin, which is implicated in the anxiety response of crayfish [35,36]. Physiological stress responses also occur in a variety of contexts associated with commercial trawling of these animals [40].

These studies on the nervous system, behaviour and stress responses are consistent with the experience of pain and fulfil 14 of the 17 criteria for pain proposed by Sneddon et al. [23]. The three not fulfilled have not been tested for decapods [24]. However, being consistent with pain is not the same as proving pain, and that is not possible for any animal taxon. Despite this lack of absolute proof, we typically employ the precautionary principle to protect animals from potential suffering [42]. Some authors have voiced opposition to the possibility of pain in decapods and seek to keep the status quo of no protection [5]. By contrast, others have accepted the possibility and have promoted the idea of protection [42,43,44]. Of note is the recent British Veterinary Association acceptance of sentience in decapods—they request that only humane slaughter methods are used [45]. Furthermore, the UK government recently commissioned an independent critical review of potential sentience and pain in decapods, but that is yet to be published. Whilst we accept that some doubt must remain, we also accept that the detailed studies cited above make it possible that decapods experience pain and thus, they should be treated in a humane manner.

## 3. Legislation Related to Decapod Protection and Welfare at the Time of Killing

In the European Union, the protection of animals at the time of killing is stipulated by the Council Regulation (EC) 1099/2009 [46]. This Regulation lays down rules for the killing of animals bred or kept for food production, wool, skin, fur, or other products, as well as the killing of animals for the purpose of depopulation and for related operations. However, for the purposes of this Regulation, the word ‘animal’ means only vertebrates (excluding reptiles and amphibians) [46]. New Zealand, Norway and Switzerland [47], however, include decapod crustaceans in their animal welfare legislation. Their decisions were based on the scientific evidence of pain and suffering, which dates as far back as the 1990s [48]. Decapods are also protected under welfare legislation in Australia [49]. France has issued a “service note” aimed at ensuring the proper management of live crustaceans and fish before sale and consumption [50]. The Swiss government, reconsidering an age-old seafood controversy (can lobsters feel pain?), has recently considered stunning and killing methods of lobsters, and has banned some practices including boiling whilst alive. As part of a wider overhaul of Swiss animal protection laws, lobsters must be now stunned before they are cooked, and must be transported in their natural environment (salt water) rather than on ice or in ice water. The government also adopted the rule that only electrical stunning or “mechanical destruction” of the brain can be permitted as stunning methods. The law, which dictates that live lobster and crayfish must be stunned before killing, was passed by the Federal Council and it came into effect in 2018 [51,52,53,54].

The practice of boiling lobsters whilst alive is also banned in New Zealand [48] and Italy [55]. In Italy, this practice is prohibited by some provincial and municipal regulations on animal protection [56]. Further, the Italian Supreme Court (III Criminal Section, sentence no. 30177/2017) has outlawed restaurants from storing lobsters on ice, arguing the practice caused animals unjustifiable suffering [57], and keeping lobsters on ice is a crime of ill-treatment (articles 727 and 544ter of Italian criminal code) because it causes suffering. The judgement balanced economic interests and a desire to reduce animal suffering [57]. By contrast, decapods are currently excluded from UK animal welfare legislation, meaning that retailers, processors, and consumers are under no obligation to consider their welfare during storage, handling, or slaughter.

## 4. Stunning and Slaughter Methods

Humane methods for slaughter are based on the general principle that the animal should be killed quickly with minimum fear, pain, or suffering [46,58]. However, many slaughter methods have been developed not to minimise stress but to achieve product quality control, production efficiency, and processor safety [59].

Crustacean slaughter techniques are diverse because different species vary greatly in their physiological and anatomical characteristics. Slaughter methods should take into account stress responses and consider metabolism, respiration, and tolerance to cerebral ganglion hypoxia. From a welfare point of view, one of the most important factors in slaughtering is the immediacy of the loss of sensibility caused by the stunning/killing method. Signs of insensibility vary with the species and the method used, but generally include: (i) no resistance to handling (e.g., the abdomen or tail can be easily extended or manipulated, and the outer mouth parts can be moved without resistance); (ii) no control of limb movement; (iii) no eye reactions when the shell is tapped; (iv) no reaction when touched around the mouthparts. Signs of stress include thrashing and autotomy (casting off body parts, such as limbs) and physiological change [57].

Before reviewing the main methods of killing decapods for use as food, we need to be clear on terminology. The term “stunning” often refers to rendering the animal insensible prior to killing, whereas the term “slaughter” is for the act of killing. However, some methods described as “stunning” result in the death of the animal. Thus, these methods should be considered “slaughter”. This is seen in electrical stunning, which is used to slaughter decapods (below). This may be contrasted with the technique of chilling, which may be used to make the animal insensible prior to a second process that kills the animal. Chilling, however, can be prolonged to ensure freezing and thus, might be used as a slaughter method. 

We have used the term “sensibility” (“insensibility”) and have attempted to avoid the term “conscious” (“unconscious”) because the latter has meanings beyond that intended in the current context [60]. An animal might respond to a variety of stimuli, but it cannot be proved that it is in a state of consciousness [60]. However, there are problems with the term “insensible”, and with the way that decapods are assessed as being insensible. For example, when decapods are chilled, they cease to respond to handling and touch, and they may be judged as being insensible. In chilling, however, the extremities will cool at a faster rate than the interior of the body. Thus, muscles in limbs might cease to function before the central ganglia stop working. Similarly, we might try to judge when an animal placed in hot water becomes insensible by assessing when it stops moving. However, the appendages of decapods are much thinner than the main body and will heat up more quickly than the interior and stop moving. In these two examples, although the animal appears to be insensible, the central ganglia might still be functional. The animal may still have some perceptual awareness of the situation and might feel pain. Thus, judging the animal as being insensible is not the endpoint for welfare concerns.

With these problems in mind, we briefly review the main techniques used in stunning/slaughter and note the advantages and disadvantages for animal welfare. These methods have been of interest to the food industry, for which humane methods of slaughter have been sought [52,61,62]. There have also been studies on the efficacy of various chemicals in killing decapods [63], but we do not cover these because they are highly unlikely to be approved in the food industry.

### 4.1. Electrical Stunning/Slaughter

An electrical stunning device called Crustastun^TM^ has been developed for the humane killing of decapods [64]. With this, the animals are stunned in a saline solution by a 110 V, 2 to 5 amp electrical charge. The device disrupts the central nervous system within a second and kills the animals within 10 s. The possibility of the device inducing stress was evaluated for crabs (*Cancer pagurus*) and lobsters (*Homarus gammarus*). After stunning, the animals were returned to a seawater container (water temperature: 10 to 12 °C), whereas sham-treated animals were placed in the Crustastun^TM^ for 10 s but without activating the electrical charge. L-lactate levels did not differ between treatments indicating that no measurable stress occurred in the Crustastun^TM^ process. It was suggested that this was due to the very rapid interruption of the neural circuits, without the possibility of recovery. Thus, Crustastun^TM^ was considered a humane method of slaughter [65], and suitable for use in restaurants immediately prior to cooking.

A large-scale dry electrical stunning system was developed by Roth and Grimsbø [65] in the project “StunCrab” (Report No. 2013; project No. 1166, March 23, 2013 and supported by Norwegian Research Council, SeaSide As, and Nofima). The aim was to develop a commercial crab stunner with 10 tons/h capacity. There were three main phases of research. Phase 1 aimed to determine the optimal electrical parameters for dry stunning, taking the animal’s impedance and position into account. Phase 2 aimed to determine the electrical setting required to stun each animal within one second and the duration required to ensure death. Phase 3 aimed to develop a commercial stunner and to test it under semi- and full-scale production. The process used a stainless steel plate, acting as one electrode, on which crabs were positioned; the other plate electrode was placed on the head of the crab. The highest impedance values for crabs were in the range of 40 to 100 Hz, for which a frequency of 50 Hz was considered adequate [65]. Exposure to 220 V, 50 Hz AC resulted in swift stunning and death within 10 s. Neither pre-chilling or keeping in air or ice water after stunning resulted in crab survival. The loss of claws or walking legs was relatively minor (3 to 6% at 220 V) and appeared to be independent of voltage or exposure time. The authors concluded that electrical stunning was adequate and humane within a large-scale commercial setting [66].

Roth and Øines [66] investigated electrical stunning and a range of other slaughter methods for edible crabs. They confirmed that electricity could render the crabs insensible within 1 s using electric field strengths of 400 V m^−1^ and more. When the exposure to the electrical current was prolonged to 10 s, a lower potential difference (220 V m^−1^) could stun the crabs. A two-stage stun with 530 V m^−1^ for 1 s, followed by 170 V m^−1^ for 2 min, induced a prolonged insensible state, but with only 60% mortality. As a consequence of stun failure, crabs exhibited a high incidence of autotomy (all appendage were lost) [67]. Nevertheless, the authors concluded that electrical stunning was the most efficient humane method to use before cooking or carving these crustaceans [66].

### 4.2. Stunning and Slaughter by Cold

Chilling in air or ice/ice slurry has been suggested for stunning decapods [67]. Crustaceans may enter a state of torpor at air temperatures of 4 °C or below [68] and may be rendered insensible when their body temperature is sufficiently reduced. Gardener [63], however, found chilling ineffective for stunning Australian giant crabs (a temperate species). When they were chilled for 14 h to 2 or 5 °C, the animals were unaffected. Furthermore, when chilled for 2 h at −1.5 °C, the crabs retained movement of the antennae and limbs, even though parts of the limbs were frozen, and they fully recovered after 45 min when returned to 10 °C [63]. 

Roth and Øines [66] considered chilling as a method of stunning edible crabs prior to boiling. Crabs chilled on ice at 0 °C still showed behavioural responses to stimuli when examined after 100 min (their internal temperature was 1.8 °C). The same authors also showed that edible crabs, placed in a freezer at −37 °C, took 30 to 40 min to lose behavioural signs of sensibility. When soaked in water at 12 °C, following 60 min in the freezer, irreversible damage was observed, and no crab recovered. The crabs typically autotomized two or more legs, indicating stress before death [66].

Weinek et al. [52] determined how cold-stunning affected neural circuits in the blue crab (*Callinectes sapidus*), the red swamp crayfish (*Procambarus clarkii*), and the white leg shrimp (*Litopenaeus vannamei*). To examine physiological changes due to cooling, these decapods were placed for 5 min in plastic boxes containing crushed sea water ice (shrimp and crabs) or freshwater ice (crayfish); the temperature in the boxes ranged between 0 and 4 °C. Immersion in ice slurry caused sedation within minutes in crayfish and shrimps, but not crabs, and a fast reduction in cardiac function was observed in shrimps. However, crabs retained a functional neural circuit over the same time, whereas shrimps and crayfish appeared to be insensible. 

Marine crustaceans should not be placed in freshwater ice slurry because this is likely to induce osmotic shock [69], and freshwater crustaceans should not be placed in a saltwater ice slurry. When a saltwater ice slurry is used for marine species, the salinity of the water in the slurry decreases as the ice melts, potentially causing osmotic shock if the animal is left in the slurry for too long. For cold-adapted species, this may occur before insensibility has been reached, unless the salinity of the slurry is maintained [70]. Furthermore, chilling in ice slurry is not recommended for temperate marine species that are adapted to low temperatures [68,70], but it might be suitable for tropical species that are more susceptible to low temperatures [69,70]. 

Chilling in air can be used for large crustaceans, but this takes longer than chilling in ice slurry because of the lower rate of heat transfer in air than in water [71]. Either method might stun crustaceans without behavioural signs of distress (such as thrashing and autotomy), but this method is not universally accepted as humane. Furthermore, there is no clear evidence that chilling in air or immersion in ice slurry results in the cessation of neural functions rather than just muscular paralysis [69]. The time required to render crustaceans insensible by air chilling varies depending on the size, species, metabolic state, and rate of chilling [70]. To be a credible, humane method of stunning, it should be shown that chilling leads to a distress-free insensible state and cessation of central neural activity; this condition should be demonstrated on a species-by-species basis [72]. Furthermore, we need to know if decapods show an aversion to low temperatures. Crayfish touched with dry ice (approximately −78 °C) did not respond more than if touched with a neutral temperature [7]; however, crayfish and lobsters do avoid low temperatures within a temperature gradient [73]. Until we have more information on responses to low temperature, we cannot evaluate the likelihood of suffering during chilling.

### 4.3. Drowning in Fresh Water

As noted above, immersing saltwater species in fresh water leads to death due to osmotic shock [68], and animals killed by this method may experience pain or distress [70]. Gardener noted that giant Australian crabs swiftly became motionless and seemed to be rigid for 10 min. However, they then became very active and autotomy was observed. The crabs also tore and pulled the walking legs and abdomen. Thus, drowning in fresh water is not suitable for rendering crabs insensible or for slaughter.

### 4.4. Salt Baths

Unlike drowning, this method involves placing the animal in a strong salt solution (35%) for a minute or less prior to boiling [74]. The treated lobsters thus showed much reduced responses when boiled. Baker [75] used a similar procedure on edible crabs and during the soaking noted reactions such as retraction of the antennae, stillness, and feeble locomotion, and spontaneous movement ceased after 10 min. When crabs were placed in boiling water, however, autotomy occurred, suggesting that neural circuits were still functioning and pain was possible [69]. Roth and Øines tried to stun edible crabs by using a salt brine of 17% NaCl or 5% KCl. These crabs showed vigorous attempts to escape and still responded to touch after 3 min. Other crabs exposed to 20% KCl did not try to escape but took 90 s to become insensitive to touch.

### 4.5. Splitting

Splitting, as a slaughter technique for lobsters, destroys the nervous system by cutting quickly along the longitudinal midline of the crustacean’s head and thorax with a large sharp knife, where the main chain of nerve centres (ganglia) is localized [69]. Splitting should result in a swift death of the animal due to the destruction of the main ganglia and thus is likely to be a humane method. However, a skilled operator is required.

### 4.6. Spiking

Spiking is commonly used for crabs, since they have more centralized nerve systems [70], comprising two anterior ganglia, rather than the chain of ganglia found in lobsters. If both ganglia are pierced and rapidly destroyed by using an awl, it should be a humane method. As with splitting, however, it should be performed by adequately trained personnel, and above all, in the proper location to ensure minimal stress and rapid brain death [69].

### 4.7. Carbon-Dioxide (CO_2_) Narcosis

It is possible to use CO_2_ to produce a deep anaesthesia and loss of consciousness after some minutes, although a study by Roth and Øines [66] suggested that CO_2_ was unsuitable for humane stunning in crabs because these crustaceans react and show behavioural signs of sensibility.

### 4.8. High-Pressure Killing

This technique, utilizing high pressure in hydrostatic pressure processors, is a commercial method to process various decapods. Their muscles become detached from the exoskeleton, and the meat may then be easily and thoroughly extracted [69,76,77]. Pressure levels around 3000 to 4200 bar (44.1 to 60.3 kpsi) are employed and the animals are held for 45 to 90 s. It is suggested that the process is suitable for either live or freshly killed animals. Live animals are said to die within 6 s but we found no data on that. The process keeps the shape of the animal and has the commercial advantage of killing microorganisms, and thus, the shelf life of the product is extended. The method is likely to become more common for commercial reasons. Whilst it seems to be a good method from a welfare perspective, more information on the speed of killing and possible suffering are required for a better evaluation.

### 4.9. Dismemberment

Some commercial practices use dismemberment of the animal without prior stunning and that action eventually kills the animal. For example, lobsters may have their claws twisted off, the eight walking legs cut off with shears, and then the tail (abdomen) meat extracted by impaling the animal on a spike. The process may take many minutes as they are passed from stage to stage and are alive for that period [78]. In other examples, crab claws are removed from the animal in a way that causes a rapid stress response [40] and the live animal is returned to the sea alive. This results in a high mortality [40], and those that survive have an impaired feeding ability [40]. Declawing has been defended by the claim that the crabs will survive and regenerate their limbs, thus producing sustainable fishery [79], but this is not borne out by experimental evidence. However, mortality is lower (but still substantial) if just one claw is removed and lower again if induced autotomy is used rather than twisting it off [80]. Even so, this is not a humane proceedure because of the problems the animals have in feeding and gaining resources [32].

### 4.10. Boiling

Boiling is a common slaughter method for decapods, but it appears to cause physiological shock and behavioural signs of aversion. For example, lobsters struggle violently for about two minutes after being placed in boiling water [1,4,68]. Lobsters and crabs thrash and try to escape, and shed their limbs, which may be signs of stress or pain (41). The main problem with boiling lobsters or crabs is the time taken for apparent death of the animal. Roth and Øines [66] measured the internal temperature of edible crabs and suggested that they can sense the heat for at least 2.5 min and prechilling could extend that time. Furthermore, chilled crabs regained their senses when placed in hot water [81]. However, the time to insensibility or death in boiling water is likely to be a function of animal size. That is, large species, such as lobsters or edible crabs, presumably take longer than small species, such as shrimps, for the central nervous system to be denatured. Thus, the duration of possible pain and stress is likely to be shorter for small species. However, whilst small species might face less suffering, they probably do not escape the welfare problem.

## 5. Conclusions

The key issue for animal welfare at the time of slaughter is potential suffering, although we accept that it is difficult to measure. Nevertheless, numerous studies have demonstrated that decapods have sensory systems that detect noxious stimuli, and sensory pathways for processing such stimuli. They have brain mechanisms that process this information and generate complex long-term behavioural responses that are not mere reflexes. Considering recent advances on possible sentience of decapods and their likely ability to experience pain, we believe the precautionary principles should be invoked in designing legislation, with the aim of minimising pain, distress, and suffering. Birch [42] described the precautionary principle as “Where there are threats of serious, negative animal welfare outcomes, lack of full scientific certainty as to the sentience of the animals in question shall not be used as a reason for postponing cost-effective measures to prevent those outcomes”. It would help if at least the minimum recommendations were issued to improve animal welfare for this order.

In terms of slaughter, the killing methods likely to be painful and distressful are: (i) any procedures that separate the thorax from the abdomen; (ii) tissue or flesh removal in live and responsive crustaceans; (iii) placing crustaceans in cold water and warming up the water to the boiling point; (iv) placing live crustaceans directly into boiling water [56]; (v) placing live marine crustaceans in fresh water. Therefore, chilling in air or ice, carbon dioxide narcosis, and “drowning” are generally not recommended methods of slaughtering crustaceans (Table 1).

Cooling on ice does not improve welfare before death [9], and Roth and Øines [66] found chilling to be slow and ineffective. Mood [69] noted that there was no evidence to suggest that chilling in the freezer/ice slurry before boiling induces anaesthesia rather than just paralysis. Nevertheless, crustaceans generally exhibit few behavioural signs of distress during chilling in ice slurry, and the method has been recommended for immobilizing and rendering animals insensible prior to slaughtering. Furthermore, some studies showed that the chilled animals died without showing signs of vigorous distress when they were subsequently immersed in boiling water. However, it has also been suggested that prior chilling might prolong the time taken to die in boiling water; therefore, the method is not acceptable [68].

Chilling in air has been applied as the initial part of a two-stage slaughter method, followed by splitting or spiking to destroy the nervous system. Decapods chilled in air do not show behavioural signs of distress. It seems that chilling helps to reduce nerve function, particularly of the appendages, and metabolic activity [68], but this might immobilize without stopping all neural function. Increasing the concentration of CO_2_ in water and changing the pH has also been used to kill decapods but this has been reported to cause distress [63,66].

Electrical stunning has been shown to cause an immediate interruption in the functions of the nervous system, which must prevent the crustacean from feeling pain or suffering distress. Accordingly, it renders crustaceans insensible in less than 1 s and, if the stun is prolonged to 5 s for lobsters or 10 s for crabs, it kills the animals [68]. It thus provides a humane way to slaughter decapods. Spiking or splitting should also result in swift death and thus might be humane.

Finally, high-pressure killing seems to be quick and thus might be a humane method for use with decapods. However, we call for more investigations of this method so that it can be fully evaluated from a welfare perspective.

Most studies on slaughter methods for decapods have focused on large species such as crabs and lobsters and small species such as shrimp and prawns have been relatively neglected. This is unfortunate because far more small decapods are used for human food than large decapods. It seems that consumers are not concerned about the possibility of suffering in small animals. This, however, is one of public opinion rather than a reaction to evidence because many of the studies that indicate possible pain have been conducted on small decapods, such as glass prawns [12] and hermit crabs [26,27,28,31]. Furthermore, prawns and shrimps are more likely to be dead (fresh, frozen, or boiled) when purchased, so consumers are not faced with the problem of killing them humanely. However, given the vast numbers of these animals that are processed for food, there is a considerable potential for suffering that is not being addressed. We have suggested that these animals would die more quickly in hot water or when chilled than lobsters, but we do not know how quick that would be or if it would represent a humane method. We urge research and legislation for such animals to better protect them by ensuring a humane death.

We note that skill and experience are required for the humane killing of crustaceans; thus, trained, competent personnel should kill these animals. Training should include appropriate handling and care for living crustaceans to reduce stress and suffering, recognition of the signs of insensibility and stress, application of the proper method of killing, and the operation and maintenance of any equipment involved in the killing process. In any case, a skilled veterinarian who is an expert in the species of interest should be consulted.

The adoption of high welfare methods might become increasingly important in achieving a high level of acceptance by consumers. Furthermore, humane slaughtering processes may contribute to higher meat quality and so could increase consumer demand [82,83]. Consumers have shown an increased interest not only in the safety and quality of food, but also in the methods of handling and killing decapods in restaurants [68], in chains of large supermarkets, and in small retail establishments. Consumers have developed an awareness of decapod biological requirements, their behavioural characteristics, and welfare. Thus, studies indicating that decapods are likely to experience pain will help policymakers to make appropriate decisions about welfare during stunning and slaughter. High quality science is expected to provide the basis of arguments to persuade European policymakers to enact and implement the relevant legislation, as in Switzerland [84].

## Figures and Tables

**Table 1:** Suitability of slaughter methods of commercially relevant decapod crustaceans.

Stunning and Killing Methods	Species	Signs of Pain and Distress	Suitable for Humane Slaughter
Electrical stunning	Lobster, Crab	None	Yes [64,65,66,69]
Splitting	Lobster	None	Yes
Spiking	Crab	None	Yes
Chilling	Large crustaceans	No behavioural signs of distress but a long time to death. Not suitable as calming before boiling.	No * [72]
Carbon-dioxide (CO_2_) narcosis	Crab	Aversive behaviour (tearing at their own sternums with their chelae, exposing internal organs) and autotomy.	No [48,63,66,67]
High-pressure killing	Lobster, Crab, other decapods	More information on the speed of killing and possible suffering are required for a better evaluation	Possibly [69,76,77]
Drowning	Saltwater species	Aversive behaviour (uncoordinated bodily movements and increased intensity of respiration) and autotomy	No [63]
Salt baths	Lobster	Abnormal behaviours (retraction of the antennae, stillness, and feeble locomotion in some cases)	No [69]
Boiling	Crab, Lobster	Physiological shock and autotomy	No [2,67,71]
Dismemberment	Lobster, Crab	Markedly decreased ability to feed, rapid stress response	No [32,40,80]

* More research is required to determine if it might be suitable for some species.
